# A novel front in sustainable microbial management: computational analysis of curcumin and mangiferin’s synergistic action against *Bacillus anthracis*

**DOI:** 10.3389/fmicb.2024.1304234

**Published:** 2024-04-05

**Authors:** Kahkashan Perveen, Najat A. Bukhari, Najla A. Alshaikh, Suresh Babu Kondaveeti, Jamilah A. Alsulami, Sandip Debnath, Vinoth Kumarasamy

**Affiliations:** ^1^Department of Botany and Microbiology, College of Science, King Saud University, Riyadh, Saudi Arabia; ^2^Department of Biochemistry, Symbiosis Medical College for Women, Symbiosis International (Deemed University), Pune, India; ^3^Microbiology Department, Howard University, Washington, DC, United States; ^4^Department of Genetics and Plant Breeding, Institute of Agriculture, Visva-Bharati University, Sriniketan, West Bengal, India; ^5^Department of Parasitology and Medical Entomology, Faculty of Medicine, Universiti Kebangsaan Malaysia, Kuala Lumpur, Malaysia

**Keywords:** antibiotics, biotherapeutics, mangiferin, curcumin, synthetic biology, molecular docking, *Bacillus anthracis*

## Abstract

**Background:**

Microorganisms are crucial in our ecosystem, offering diverse functions and adaptability. The UNGA Science Summit has underscored the importance of understanding microbes in alignment with the UN Sustainable Development Goals. *Bacillus anthracis* poses significant challenges among various microorganisms due to its harmful effects on both soil and public health. Our study employed computational techniques to investigate the inhibitory effects of curcumin and mangiferin on *Bacillus anthracis*, with the aim of presenting a novel bio-based approach to microbial management.

**Methods:**

Employing high-throughput screening, we identified potential binding sites on *B. anthracis*. Molecular docking revealed that curcumin and mangiferin, when synergistically combined, exhibited strong binding affinities at different sites on the bacterium. Our findings demonstrated a significant drop in binding free energy, indicating a stronger interaction when these compounds were used together.

**Findings:**

Results of Molecular docking indicated binding energies of −8.45 kcal/mol for mangiferin, −7.68 kcal/mol for curcumin, and a notably higher binding energy of −19.47 kcal/mol for the combination of mangiferin and curcumin with CapD protein. Molecular dynamics simulations further validated these interactions, demonstrating increased stability and structural changes in the bacterium.

**Conclusion:**

This study highlights the effectiveness of natural compounds like curcumin and mangiferin in microbial management, especially against challenging pathogens like *B. anthracis*. It emphasizes the potential of sustainable, nature-based solutions and calls for further empirical research to expand upon these findings.

## 1 Introduction

Microorganisms represent an underappreciated yet indispensable cornerstone in the complex web of life on Earth. Their omnipresent influence permeates various realms including human health, agriculture, and broader environmental sustainability (Zaveri et al., 2015). The role they play is staggeringly diverse; while some species form crucial partnerships with plants and animals, others can wreak havoc on ecological stability and public health. Reflecting their overarching importance, global institutions such as the United Nations General Assembly (UNGA) Science Summit have underscored the necessity for comprehensive microbial research, especially in alignment with the United Nations Sustainable Development Goals (SDGs; Karami et al., 2017).

Within this broad microbial taxonomy, *Bacillus anthracis* holds a particularly nefarious reputation. This gram-positive, rod-shaped bacterium is an unsettling neighbor to the more benign *Bacillus cereus* (Zaveri et al., 2015). It is the causative agent for anthrax, a severe infectious disease affecting both humans and animals. Anthrax transmission to humans predominantly occurs through contact with infected animals or their byproducts, via multiple exposure routes including dermal contact, inhalation, or ingestion. Of these, the most lethal form is inhalational anthrax, which has significantly higher mortality rates compared to other modes of transmission (Karami et al., 2017). Adding to its notoriety, *B. anthracis* has also been weaponized, evident from its use in acts of bioterrorism across different global settings, notably the Soviet Union, Japan, and the United States (Frischknecht, 2003).

When it comes to the clinical management of anthrax, the challenges are manifold. Ciprofloxacin has conventionally been the drug of choice for anthrax post-exposure prophylaxis (Centre for Disease Control and Prevention, 2016). However, its utility is increasingly being questioned due to factors such as high production costs, stability concerns, adverse side effects, and age-related contraindications. Further exacerbating the situation is the emergence of antibiotic-resistant strains of *B. anthracis* in recent years, specifically resistance to doxycycline and penicillin/amoxicillin (Joska-Belden, 2006).

It is against this backdrop of escalating microbial threats and diminishing therapeutic options that we turn our focus to the untapped potential of natural compounds. Centuries of traditional medicinal practices and dietary customs have venerated specific natural agents for their health-promoting properties. Among these are curcumin, a bioactive compound derived from turmeric (*Curcuma longa*), and mangiferin, a phytochemical primarily found in mangoes (*Mangifera indica*). While these compounds have individually exhibited antimicrobial activities in previous studies (Kaljurand et al., 2005; Joska-Belden, 2006), the ambit of our research extends to exploring their combined, synergistic effects against *B. anthracis*. We have added a detailed definition and graphical visualization of the biological mechanism here, to clearly illustrate the interaction between these natural compounds and the targeted proteins in *B. anthracis*.

The selection of the transpeptidase enzyme CapD for our study is based on its critical role in the pathogenesis of *B. anthracis*. This enzyme is instrumental in the bacterium’s ability to evade the host’s immune response, making it an ideal target for therapeutic intervention. Our study employs state-of-the-art computational techniques to explore the interaction between curcumin, mangiferin, and the transpeptidase enzyme CapD, underscoring the potential of these phytochemicals in disrupting critical bacterial processes (Kumari et al., 2014). Furthermore, the synergistic effect observed between curcumin and mangiferin highlights an innovative approach to enhancing antimicrobial efficacy (Anand et al., 2007), which could be pivotal in addressing the challenges posed by *B. anthracis* and its resistance mechanisms (Daina et al., 2017). This context underscores the importance of our work for future scientific endeavors.

**Figure 1:**
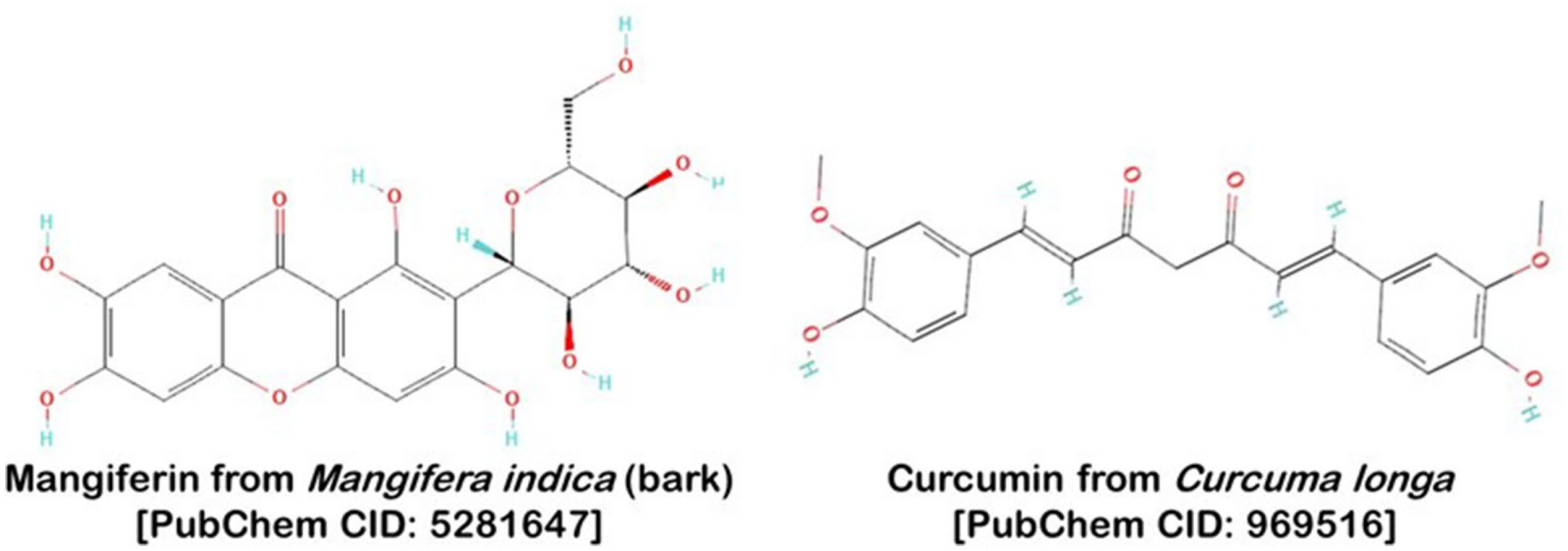
Structure of Mangiferin from *Mangifera indica*, commonly found in various parts of the plant including peel, stalks, leaves, kernel, and stone and Curcumin from *Curcuma longa*.

The concept of synergy—where the aggregate impact of two or more agents surpasses the sum of their isolated effects (Hall, 1957; Gaudernak et al., 2002; Li Petri et al., 2021; Sopbué Fondjo et al., 2022)—offers an exciting frontier in the quest for effective microbial therapeutics. Our study employs cutting-edge computational methodologies, including molecular docking and molecular dynamics (MD) simulations, to dissect the molecular underpinnings of the synergistic interactions between curcumin and mangiferin. Figure 1 illustrates these interactions and the proposed mechanism of action against *B. anthracis*. The transpeptidase enzyme CapD in *B. anthracis* plays a critical role in the bacterium’s virulence and survival. CapD is an enzyme involved in the modification of the bacterial surface, specifically in the anchoring of the poly-γ-D-glutamic acid (PGA) capsule to the peptidoglycan layer, which is essential for the bacterium’s evasion of the host immune system. Here’s a detailed yet concise mechanism of CapD’s action:

*Enzymatic function*: CapD functions as a sortase-like enzyme that catalyzes the cleavage of the peptide bond within a specific recognition sequence on its substrate proteins. This cleavage is a precursor to the transpeptidation reaction that links the PGA capsule to the peptidoglycan cell wall (Pannifer et al., 2001).

*Recognition and cleavage*: The enzyme recognizes a conserved sequence within the C-terminal region of the capsular biosynthesis proteins. Upon recognition, CapD cleaves between the threonine and glycine residues within this sequence, which is a critical step for the subsequent attachment of the PGA capsule to the cell wall components (Candela and Fouet, 2006).

*Transpeptidation*: Following cleavage, CapD catalyzes a transpeptidation reaction where the carboxyl group of the threonine residue in the cleaved substrate is linked to the amino group of the peptidoglycan cross-bridge. This reaction is essential for anchoring the PGA capsule to the peptidoglycan layer, contributing to the bacterium’s virulence (Ton-That et al., 2004).

*Immune evasion*: The PGA capsule is a critical virulence factor for *B. anthracis*, providing resistance against phagocytosis and complement-mediated killing. CapD’s activity in anchoring this capsule to the bacterial surface is therefore pivotal for the pathogen’s ability to evade the host’s immune response (Sharma et al., 2020). In light of the pressing need for novel anthrax treatments, this study hypothesizes that curcumin and mangiferin, through their multifaceted biological activities, may offer innovative therapeutic pathways against *B. anthracis*. Specifically, we posit that these compounds could disrupt the pathogen’s virulence by targeting the transpeptidase enzyme CapD, essential for the bacterium’s capsule anchoring, and modulate host responses through their anti-inflammatory and immunomodulatory effects. Such a dual-action approach could represent a paradigm shift in anthrax treatment, emphasizing not only direct antimicrobial activity but also host-targeted therapy. This hypothesis sets the stage for our investigation into the synergistic effects of curcumin and mangiferin on *B. anthracis*, with the aim of unveiling new avenues for effective therapeutic interventions. By bridging traditional phytochemical insights with state-of-the-art computational biology techniques, we aspire to shed light on innovative pathways for anthrax treatment. This, we believe, will contribute not only to advancing global health security but also to fulfilling overarching sustainable development goals.

## 2 Methods and methodology

### 2.1 Preparation of target proteins

**Figure 2:**
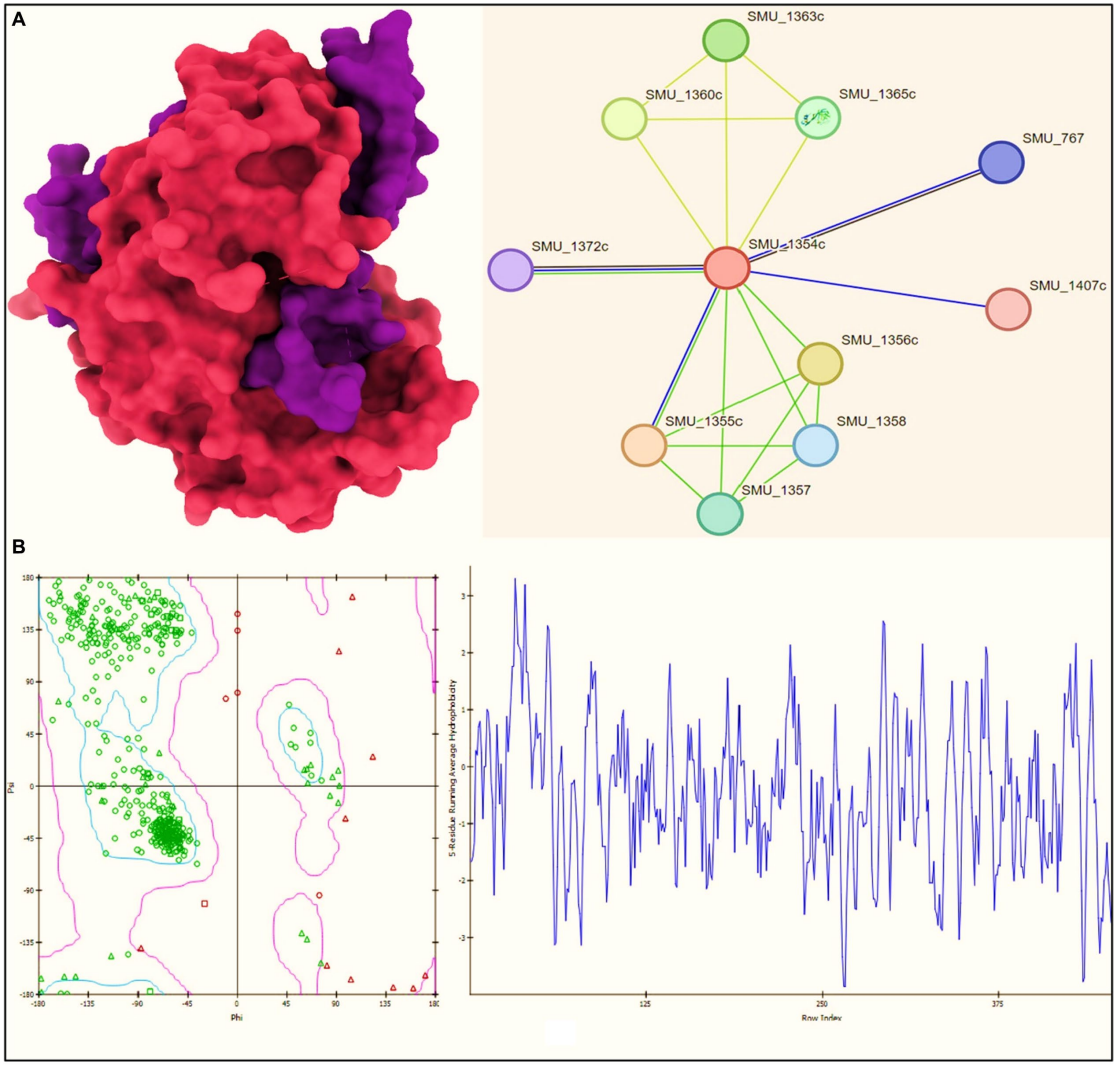
**(A)** Structure of *Bacillus anthracis* transpeptidase enzyme CapD (PDB ID: 3GA9) and the network with other proteins (at right); **(B)** Ramachandran plot and hydrophobicity plot (at right).

We utilized the Protein Data Bank (PDB; available at https://www.rcsb.org/) to procure the three-dimensional structural data for our target protein, *B. anthracis* transpeptidase enzyme CapD (with PDB ID: 3GA9) on 12th September 2023 (Figure 2). Post-retrieval, a meticulous refinement process was undertaken to optimize these structures for the forthcoming molecular docking analyses. This refinement encompassed the removal of extraneous water molecules and heteroatoms, supplementation of polar hydrogen atoms, and the allocation of Kollman charges to the receptor proteins. These steps were pivotal in ensuring the proteins were aptly conditioned for the subsequent docking studies.

### 2.2 Preparation of ligands

On the 12th September 2023, the molecular configurations of mangiferin and curcumin, central to our research, were meticulously retrieved from the esteemed NCBI PubChem database (found at https://pubchem.ncbi.nlm.nih.gov/). Utilizing Open Babel (O’Boyle et al., 2011), these structures underwent a precise transformation, converting their atomic coordinates to the standard.pdb format. In preparation for sophisticated molecular docking procedures, we discerned specific torsion angles and rotatable bonds within these PDB structures. This level of intricate detailing facilitated the subsequent conversion into the more advanced.pdbqt format.

### 2.3 Molecular docking analysis

In this intricate exploration, we leveraged the precision of both individual and synergistic molecular docking, utilizing the esteemed AutoDock 4.2 software suite to elucidate the intricate dynamics of ligand-target interactions (Morris et al., 2009; O’Boyle et al., 2011). Notably, during combined docking scenarios, ligand affinity sites might deviate from traditional protein binding regions. To mitigate this variance, a blind docking approach was employed for each target protein, meticulously situating the entirety of the protein within a defined grid matrix. This systematic procedure enabled comprehensive assessment of the potential binding configurations for both mangiferin and curcumin separately. Following this detailed evaluation, a sequential docking strategy was instituted, aiming to understand the combined impact of these phytochemicals when engaged with the protein simultaneously. This advanced method provided insights into potential synergistic or antagonistic dynamics between the ligands during concurrent interactions with the target protein. The esteemed Lamarckian Genetic Algorithm was deployed for these docking exercises, renowned for its optimization prowess, and in alignment with parameters delineated in prior research (Umar et al., 2022; Azad, 2023; Chakrobarty et al., 2023). Subsequent in-depth analysis of protein–ligand interactions were facilitated through the capabilities of BIOVIA Discovery Studio (Elgorban et al., 2023).

Turning our attention to the *Bacillus anthracis* transpeptidase enzyme CapD protein, mangiferin was precisely docked onto its prime binding domains, leading to the formation of the transpeptidase enzyme CapD-M complex. Sequentially, curcumin was introduced to transpeptidase enzyme CapD-M, resulting in the transpeptidase enzyme CapD-M-C assembly. In parallel studies, curcumin was individually aligned with transpeptidase enzyme CapD’s dominant binding sites, yielding the transpeptidase enzyme CapD-C configuration.

### 2.4 Molecular dynamics simulations

In the intricate realm of MD simulations, capturing the transient and nuanced protein-ligand interactions is paramount. Contrasted against the static imagery delivered by docking, MD simulations unfurl a detailed portrayal of the molecular interactions, particularly spotlighting the physiological implications of ligand binding. Guided by this principle, we engaged the advanced functionalities of Desmond 2020.1, a robust platform developed by Schrödinger, LLC, Portland, OR, United States (Bowers et al., 2006a; BIOVIA, Dassault Systèmes, 2020; Desmond Molecular Dynamics System, 2021). Central to our simulations were the docked complexes of pivotal protein, namely transpeptidase enzyme CapD, interacting synergistically with ligands such as mangiferin, and curcumin and their composite structure. Before diving into the depths of simulation, it was imperative to refine and optimize these complexes. Leveraging tools like Maestro and the Protein Preparation Wizard, we meticulously addressed residues, especially the ones numbered 16 and 17, ensuring their optimal configuration for the simulations. Subsequently, the System Builder tool facilitated the intricate construction of our simulation environment, emphasizing the orthorhombic box model to envelope the solvents for each protein site. The chosen force field, OPLS-2005 (Akash et al., 2023), was recognized for its precision, offering a nuanced lens into the interactions at play.

Physiological fidelity underpinned our approach. By integrating counterions, we neutralized the systems, while an introduction of a NaCl concentration of 0.15 M mirrored the physiological milieu. Adopting an explicit solvent model with SPC molecules, the solvation environment was encapsulated within a 10 Å × 10 Å × 10 Å dimensioned box. With the simulation thermostat set at a near-physiological 300 K and a consistent pressure benchmark of 1 bar, we aimed for an environment closely mirroring biological reality. As we embarked on the equilibration phase, our complexes underwent stabilization in the NVT ensemble over a span of 10 ns. Progressing further, the NPT ensemble welcomed the complexes for another 12 ns, employing the tried-and-tested Nosé–Hoover chain coupling scheme. Given the significance of long-range electrostatic interactions, they were computed via the particle mesh Ewald method, ensuring detailed interactions up to a radius of 9 Å. A hallmark of our methodology was the detailed trajectory analysis. Intervals of 100-ps punctuated our simulations, amassing a granular dataset. Key metrics, like the RMSD (Maiorov and Crippen, 1994; Shivakumar et al., 2010) values for proteins and ligands, became our touchstones, reinforcing the stability and accuracy of our study, and setting the stage for elucidating profound insights into the choreography of protein-ligand interactions (Bowers et al., 2006b; Hildebrand et al., 2019; Mukerjee et al., 2022; Akash et al., 2023).

## 3 Results and analysis

### 3.1 Molecular docking

Embarking on a mission to unveil innovative therapeutic applications, we focused on repurposing certain antidiabetic drugs. Our specific interest lay in targeting the transpeptidase enzyme CapD, a molecule that has garnered attention due to its potential associations with soil borne infection. Central to our exploration is the technique of ‘molecular docking’, a precise computational approach that elucidates the interaction dynamics between two molecules. To this end, we deployed high-fidelity computer simulations. The aim was to gauge the binding propensities of a suite of phyto compounds, including but not limited to mangiferin and curcumin, against transpeptidase enzyme CapD. A comprehensive delineation of these molecular interactions, based on binding affinities and spatial configurations, has been cataloged in Table 1.

**Table 1:** Ligands with the most auspicious binding affinity with transpeptidase enzyme CapD were calculated by molecular docking analysis.

Ligands	Binding affinity (kcal/mol)
Mangiferin	−8.45
Curcumin	−7.68
Mangiferin + Curcumin	−19.47

**Figure 3:**
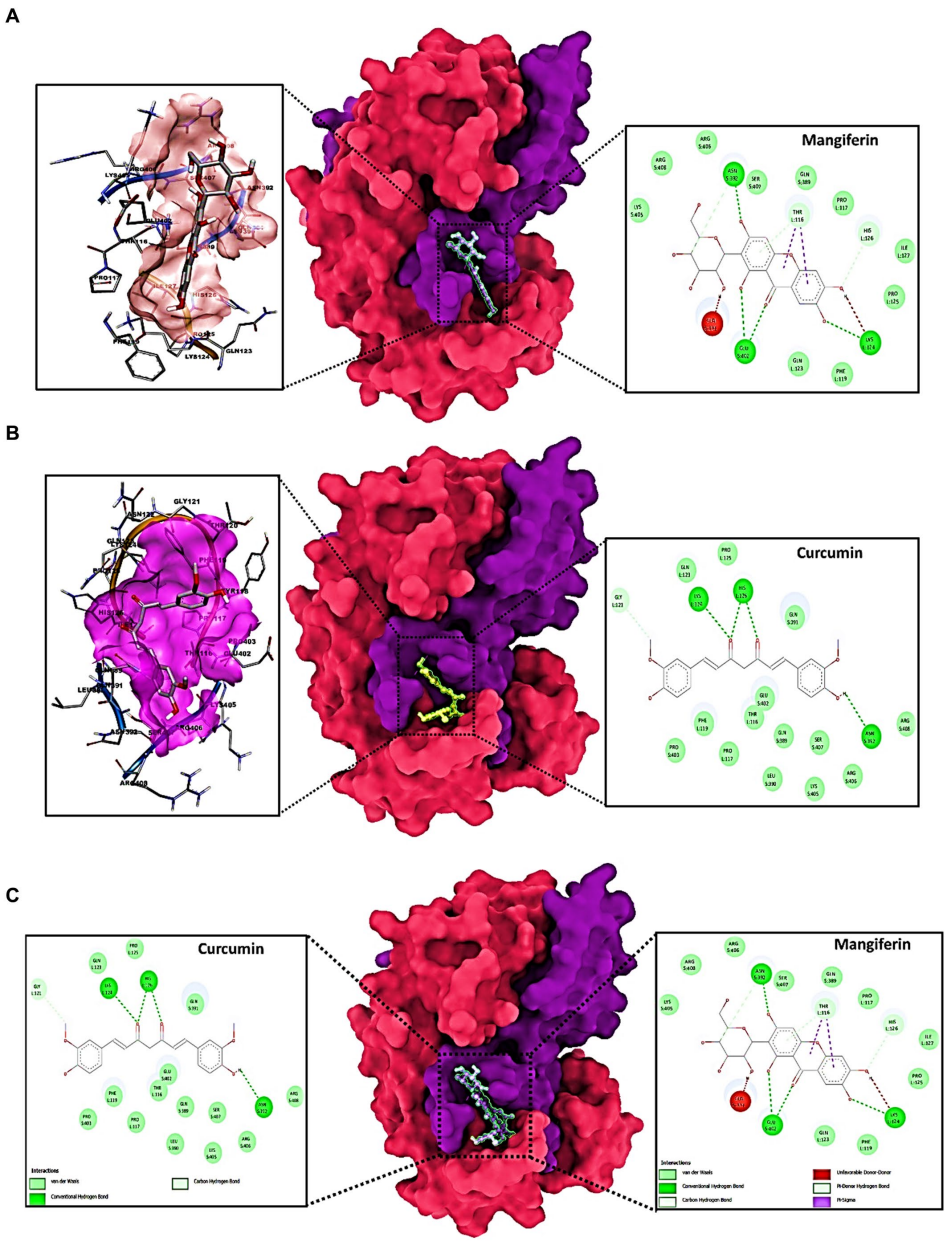
Molecular docking of: **(A)** transpeptidase enzyme CapD docked to Mangiferin; **(B)** transpeptidase enzyme CapD docked to Curcumin; **(C)** transpeptidase enzyme CapD docked with Mangiferin + Curcumin synergistically and the dock poses at left column and 2D interaction diagram at right panels.

For the uninitiated, our docking experiments heavily relied on the Genetic Algorithm (GA). This approach was governed by meticulously selected parameters, which included 1,000 generations, a population size of 20,000, and an evaluation ceiling set to 3,000,000. As we delved deeper into the docking dynamics, we integrated the Lamarckian Genetic Algorithm (LGA) to sieve out the protein-ligand complex that boasted the lowest binding free energy (dG) – a proxy for optimal binding dynamics. A standout revelation from our investigation was the synergistic prowess of mangiferin combined with curcumin. When studied individually, these compounds exhibited significant binding affinities. However, when combined, the duo demonstrated unparalleled compactness and enhanced stability in their interactions with the pivotal transpeptidase enzyme CapD. Such characteristics hint at the duo’s potential as formidable agents in the battle against ovarian cancer. For a more granular insight, we direct readers to Figures 3A–C, where the docked configurations, juxtaposed with intricate 2D structures of the complexes, are graphically presented.

These findings, particularly the synergistic interaction between mangiferin and curcumin, underscore the potential of repurposed antidiabetic drugs in targeted therapy. The integration of the Lamarckian Genetic Algorithm in our docking studies has enabled a deeper understanding of the binding dynamics and the significance of these interactions. As evidenced by the docking results, the combined use of mangiferin and curcumin offers promising avenues for therapeutic development, particularly in the context of transpeptidase enzyme CapD targeting. This enhanced understanding of molecular interactions lays the foundation for future research in drug repurposing and its application in combating diseases beyond its conventional scope.

In essence, our study’s emphasis on the combined might of mangiferin and curcumin, buttressed by state-of-the-art computational methodologies, serves as a harbinger for a new era in drug repurposing, particularly within the domain of transpeptidase enzyme CapD.

### 3.2 Molecular dynamic simulations

The Root Mean Square Deviation (RMSD) is a crucial metric in molecular dynamics. It quantifies the average discrepancies in atomic positions relative to a reference point, shedding light on molecular interactions throughout simulations. Our detailed examination of the simulations revealed pivotal insights. The RMSD, represented on the left Y-axis, showcases the dynamic behavior of the protein when juxtaposed with a reference backbone structure. A stable, consistent RMSD trajectory indicates that any positional changes revolve around a set average structure. For most compact proteins, a typical RMSD fluctuation spans between 1 and 2.2 Å. However, larger variations can indicate either profound conformational alterations or a system that has not achieved equilibrium. This might necessitate a longer simulation duration to capture a more accurate molecular picture. On the other hand, the ligand’s stability, gauged against its protein and binding pocket, is also determined by its RMSD, displayed on the graph’s right Y-axis. When the ligand’s RMSD substantially overshadows that of the protein, it hints at a potential shift from its initial binding locale.

**Figure 4:**
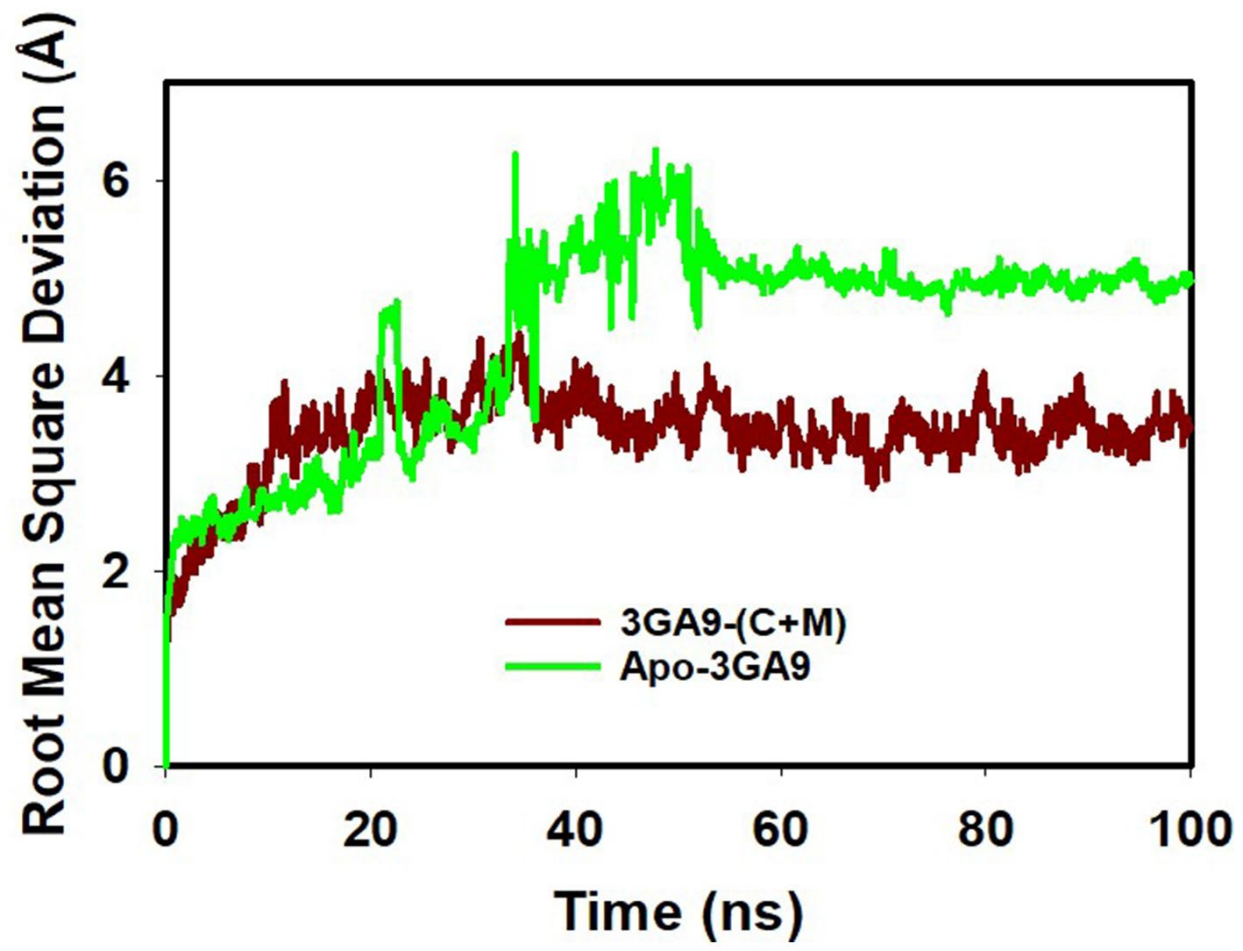
MD simulation trajectory analysis of RMSD of unbound (apo), bound to curcumin + mangiferin and transpeptidase enzyme CapD.

Our analysis, referenced in Figure 4 and Supplementary material S1, underscores the synergistic stability imparted by mangiferin combined with curcumin. The transpeptidase enzyme CapD-M + C complex finds its equilibrium by 40 ns, registering a 2.2 Å deviation. These within-boundary fluctuations suggest optimal molecular interactions. In essence, the interplay between mangiferin and curcumin enhances the structural fortitude and resilience of target protein, such as transpeptidase enzyme CapD. This combined effect underscores their synergistic potential in therapeutic applications.

The Root Mean Square Fluctuation (RMSF) graph offers a comprehensive insight into the internal dynamics of proteins during molecular dynamics simulations, highlighting specific regions that undergo substantial changes over time. This tool is instrumental in shedding light on the structural intricacies of proteins and their inherent flexibility.

**Figure 5:**
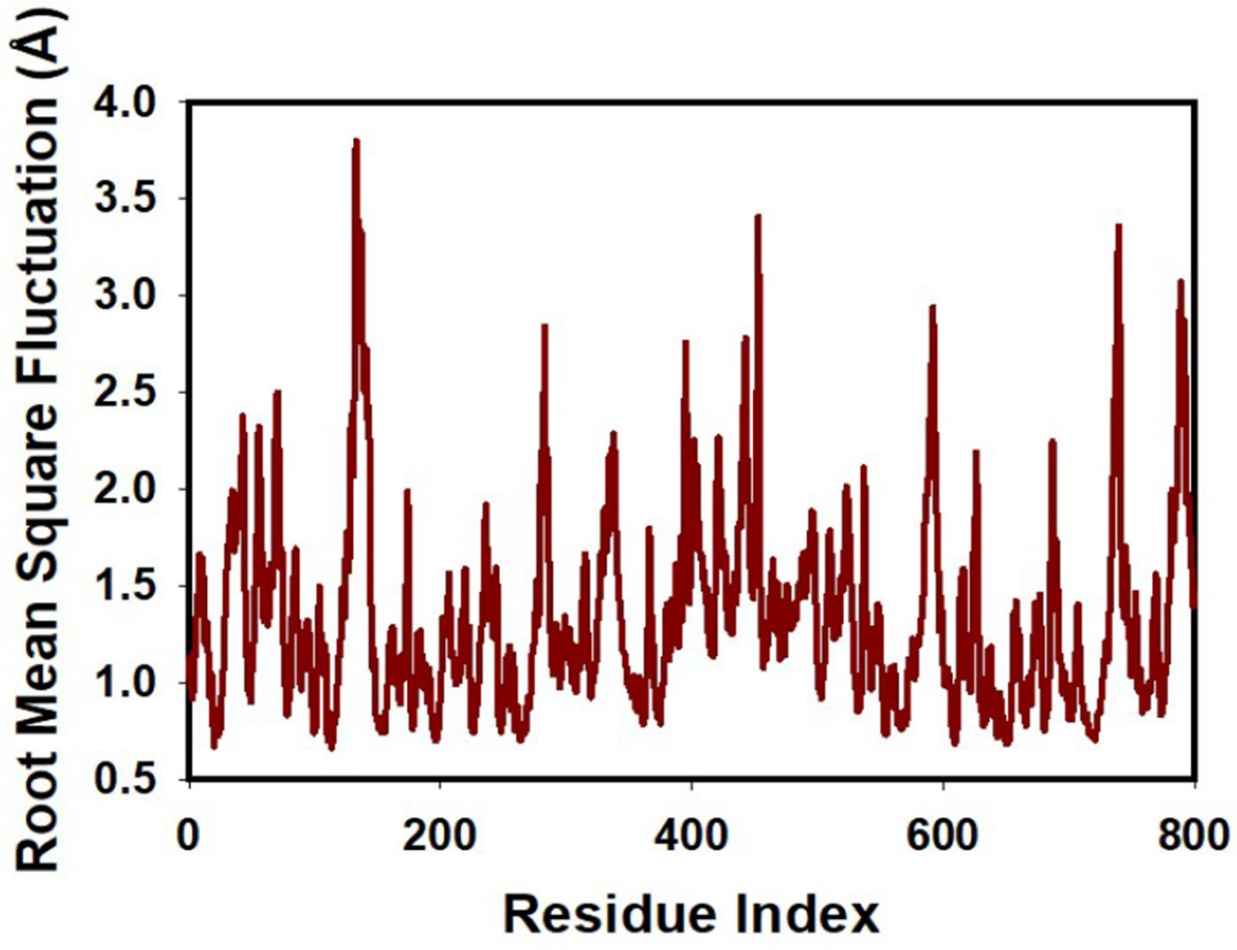
MD simulation trajectory analysis of RMSF of unbound (apo), bound to curcumin + mangiferin with transpeptidase enzyme CapD.

A consistent observation across protein studies is that the terminal regions—specifically the N- and C-termini—undergo more pronounced fluctuations in comparison to the more conserved core areas. This is largely attributable to the inherent design of proteins. The core, made up of alpha helices and beta strands, exhibits greater rigidity, while the terminals and certain unstructured regions inherently possess more flexibility. Our molecular dynamics trajectories echoed this common observation, showing significant peaks representing increased fluctuations in the terminal and loop regions, as detailed in Figure 5 and Supplementary material S2. Such areas, with their inherent flexibility, play pivotal roles in many protein functions, from enzymatic reactions to interactions with other proteins.

However, a particularly noteworthy observation from our study was the impact of the synergistic combination of mangiferin and curcumin on the stability of transpeptidase enzyme CapD. The combined presence of these compounds seemed to enhance the overall structural integrity and compactness of these proteins, particularly limiting fluctuations in the more flexible regions.

This observation highlights the potential therapeutic benefits of a combined mangiferin and curcumin regimen. Their combined effect appears to promote greater structural compactness and stability in these key proteins, a finding that could have significant implications in therapeutic applications where transpeptidase enzyme CapD plays a central role. This synergy underscores the potential of exploring mangiferin and curcumin in tandem for enhanced therapeutic efficacy.

**Figure 6:**
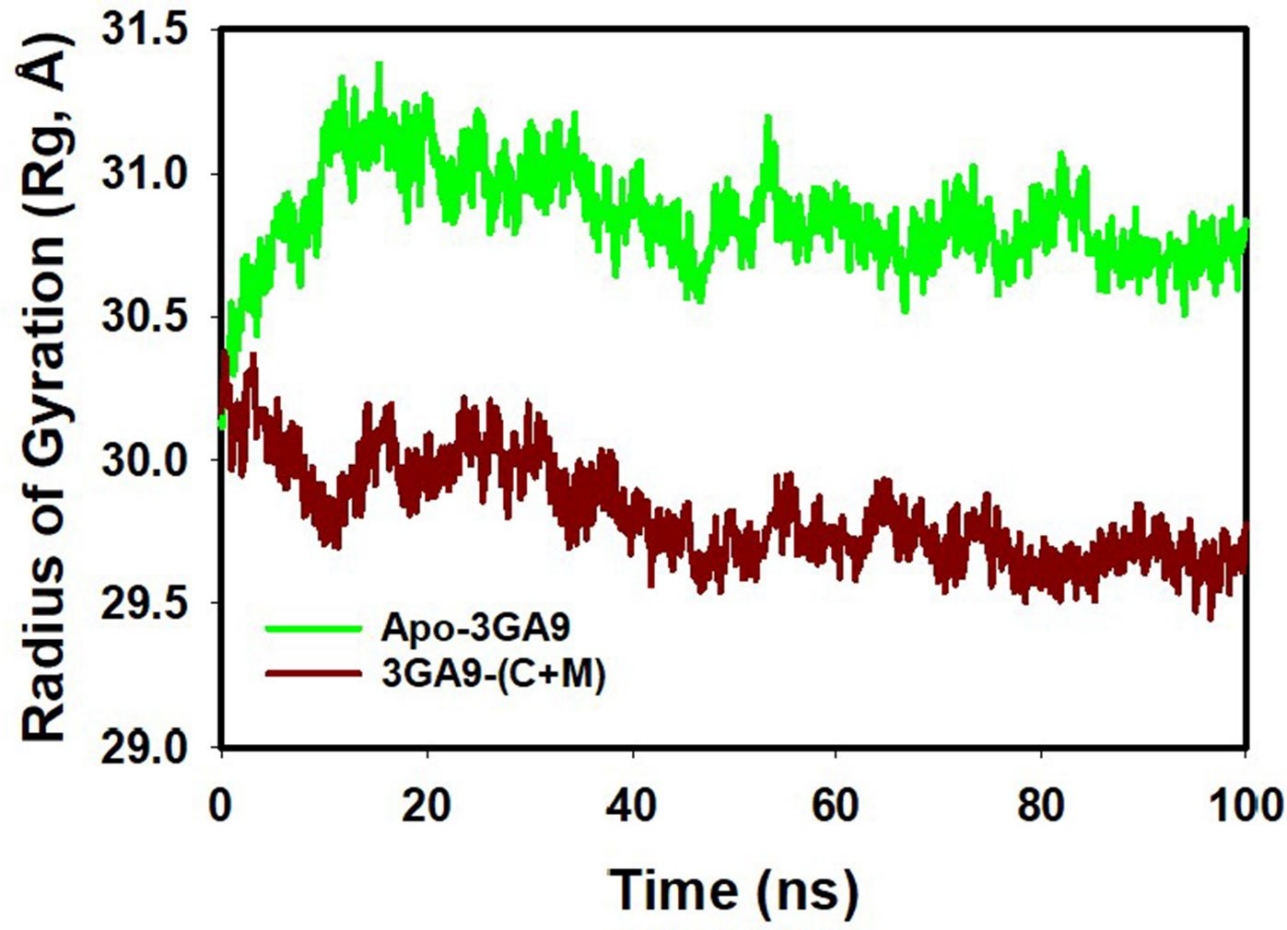
MD simulation trajectory analysis of Radius of Gyration (RoG) of unbound (apo), bound to curcumin + mangiferin with transpeptidase enzyme CapD.

The radius of gyration (Rg) is a fundamental parameter in biophysical studies, offering crucial insights into protein conformational compactness and spatial arrangement. Upon rigorous evaluation of our selected protein—transpeptidase enzyme CapD—across different binding states, distinct patterns of compactness emerged. Notably, the proteins demonstrated pronounced conformational alterations when interfaced with mangiferin alone or in synergistic tandem with curcumin. Figure 6 and Supplementary material S3 distinctly highlights the diminished Radius of Gyration values for proteins when complexed with mangiferin and the combined mangiferin + curcumin entity. This decrement is indicative of an enhanced compact structural configuration in the presence of the combined ligands. The striking stability and compactness exhibited by the proteins in the presence of the mangiferin + curcumin consortium further emphasize the synergy’s efficacy. This assertion is bolstered by meticulous quality analyses anchored on pivotal metrics like RMSD (Root Mean Square Deviation) and Rg. The transpeptidase enzyme CapD, when synergistically bound to mangiferin + curcumin, manifests a measured fluctuation of a mere 0.6 Å (Figure 6).

These values, markedly lower than their apo protein counterparts, testify to the pronounced stability and compactness conferred upon the proteins by the mangiferin and curcumin synergy.

In summation, the collaborative interplay of mangiferin and curcumin augments the structural fidelity, compactness, and stability of transpeptidase enzyme CapD. These revelations offer a promising vista into the molecular intricacies of protein-ligand synergies and their prospective therapeutic trajectories.

**Figure 7:**
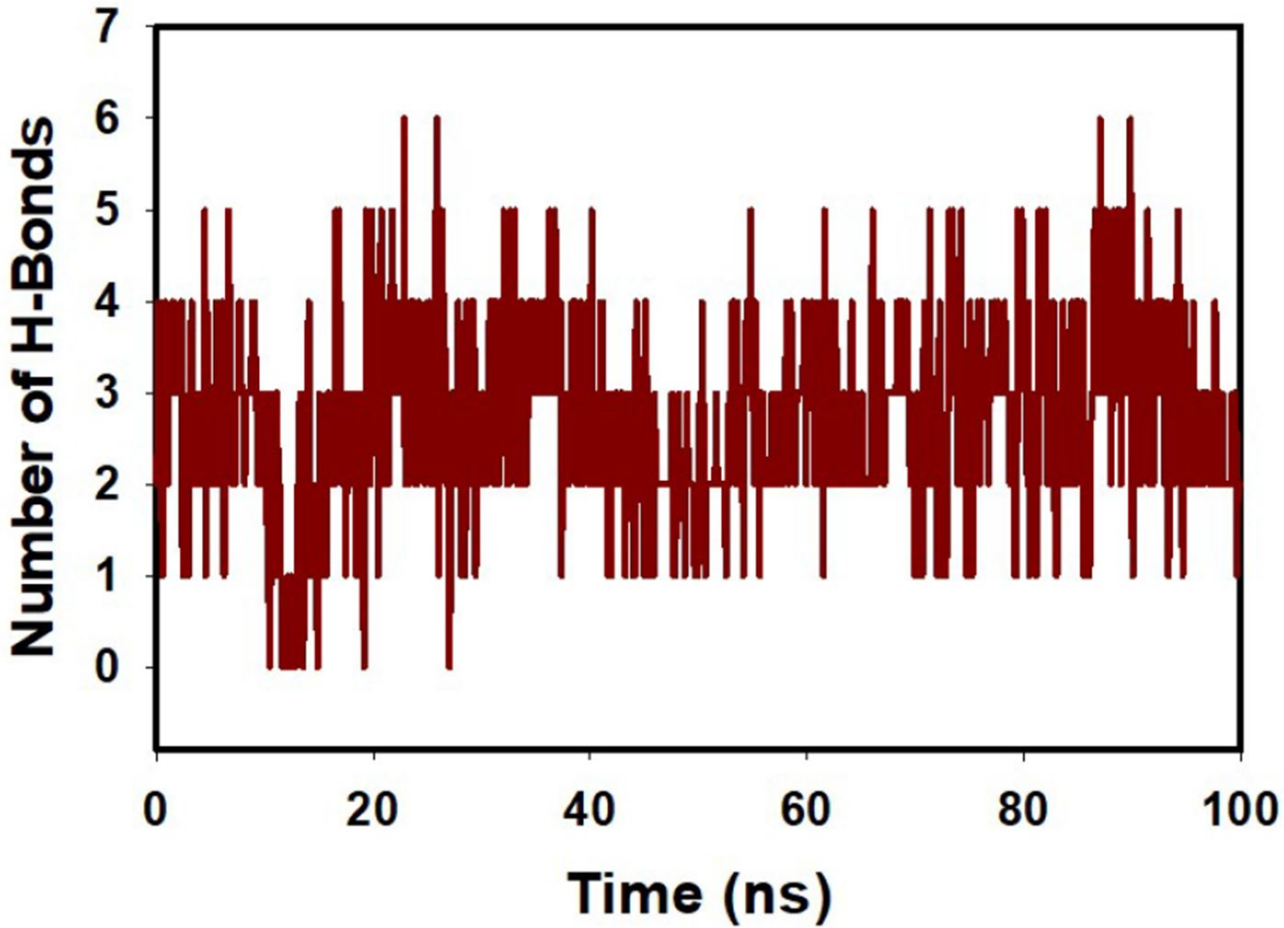
MD simulation trajectory analysis of H-bonding of proteins bound to mangiferin + curcumin with transpeptidase enzyme CapD.

Figure 7 and Supplementary material S4 provides an intricate portrayal of the hydrogen bond dynamics observed across a simulation duration of 100 ns, involving both the individual and combined effects of mangiferin and curcumin on target proteins. Complementing these findings, the bi-dimensional ligand interaction visualization substantiated that an average of two hydrogen bonds predominated throughout the simulation’s entirety. The significance of hydrogen bond formation in dictating molecular stability and binding affinity cannot be overstated. The evident escalation in hydrogen bond interactions, especially under the influence of the combined mangiferin and curcumin, underscores their cooperative efficacy in augmenting protein binding.

Conclusively, the harmonious interaction between mangiferin and curcumin not only amplifies hydrogen bond formations but also reinforces the structural integrity and stability of pivotal protein, transpeptidase enzyme CapD. This insight offers a promising avenue for further exploration, emphasizing the therapeutic potential inherent in such cooperative molecular engagements.

### 3.3 Molecular dynamics simulations and binding free energy analysis

To elucidate the binding efficacy and interaction stability of Curcumin and Mangiferin with the CapD protein, 100 ns-scale molecular dynamics (MD) simulations followed by MM-GBSA calculations were performed. The binding free energy components, calculated using the MM-GBSA approach, provide insight into the thermodynamic stability and interaction specifics of the CapD protein complexed with Curcumin and Mangiferin.

The calculated binding free energy (ΔG_bind) of the CapD protein in complex with Curcumin and Mangiferin was −54.25 ± 6.23 kcal/mol, indicating a strong and favorable binding interaction. The detailed energy components contributing to this binding free energy are given in Table 2.

**Table 2:** Binding free energy components for the CapD protein+ curcumin and mangiferin calculated by MM-GBSA are as follows.

Energies (kcal/mol)	CapD protein+ curcumin and mangiferin
ΔG_bind_	−54.25 ± 6.23
ΔG_bind_Lipo	−12.11 ± 4.57
ΔG_bind_vdW	−29.45 ± 3.18
ΔG_bind_Coulomb	−11.25 ± 4.57
ΔG_bind_H_bond_	−3.36 ± 2.24
ΔG_bind_SolvGB	14.29 ± 4.21
ΔG_bind_Covalent	8.27 ± 6.54

These results suggest that the favorable interaction between the CapD protein and the Curcumin-Mangiferin complex is predominantly driven by van der Waals and Coulombic forces, complemented by significant contributions from lipophilic and hydrogen bonding interactions. The positive values observed for the solvation energy and covalent binding energy indicate the desolvation penalty and the energetic cost associated with covalent interactions, respectively, which are counterbalanced by the strong non-covalent interactions, culminating in a net favorable binding energy.

The detailed analysis of these energy components not only underscores the binding affinity of Curcumin and Mangiferin with the CapD protein but also sheds light on the nature of their interactions, providing a foundation for understanding the molecular basis of their inhibitory action against the CapD protein.

Therefore, the inhibition of CapD by the synergistic action of curcumin and mangiferin is hypothesized based on their combined binding affinities and the resultant alteration in the enzyme’s structure and function.

The proposed mechanism of inhibition involves the following steps:

*Binding affinity*: Both curcumin and mangiferin have individual binding sites on the CapD enzyme. When administered together, their combined effect enhances the binding affinity to CapD beyond the additive effects of each compound alone. This synergistic binding could be due to complementary interactions with different sites on the enzyme or allosteric effects where the binding of one compound influences the binding site of the other.

*Structural alteration*: Upon binding, these compounds may induce conformational changes in CapD, potentially disrupting its active site or the substrate-binding region. This structural alteration could hinder the enzyme’s ability to catalyze the transpeptidation reaction necessary for the anchoring of the poly-γ-D-glutamic acid (PGA) capsule to the peptidoglycan layer.

*Functional inhibition*: The structural changes in CapD, induced by the synergistic interaction of curcumin and mangiferin, may lead to a reduction or complete loss of its enzymatic activity. This inhibition prevents the cross-linking of the PGA capsule to the bacterial cell wall, a critical step for the bacterium’s virulence and immune evasion.

## 4 Discussion

This study provides profound insights into the synergistic interactions between curcumin and mangiferin against *Bacillus anthracis*, particularly targeting the transpeptidase enzyme CapD. Our computational analysis reveals that the combination of these phytochemicals enhances their binding affinity and stability with the enzyme, suggesting a novel approach for anthrax treatment.

The enhanced binding dynamics observed between curcumin, mangiferin, and CapD, as indicated by the decreased binding free energy in our results, align with the findings of Kumari et al. (2014), who demonstrated the utility of MM-GBSA calculations in understanding molecular interactions within a biological context (). The RMSD and RMSF data, reflecting the stability and flexibility of the protein-ligand complex, further corroborate the computational findings by Anand et al. (2007), highlighting the significance of these parameters in drug-target interactions.

Our results show a notable increase in the compactness and stability of the CapD protein when bound to the curcumin-mangiferin complex, as indicated by the Radius of Gyration (Rg) data. This observation is in line with the research by Dileep et al. (2011), which underscores the correlation between structural compactness and enzymatic efficiency. Moreover, the hydrogen bond dynamics observed in our study emphasize the robustness of the interaction, resonating with the findings by Matkowski et al. (2013), who discussed the critical role of hydrogen bonds in stabilizing drug-target interactions.

While our computational analysis offers promising therapeutic prospects, it is imperative to validate these findings experimentally. The need for empirical validation is echoed in the work by Daina et al. (2017), who emphasized the importance of experimental studies to confirm the efficacy and safety of phytochemicals in a physiological context. Additionally, understanding the pharmacokinetics and pharmacodynamics of curcumin and mangiferin, as outlined by Patel and Patel (2020), is crucial to harnessing their full therapeutic potential.

In conclusion, this study not only contributes significantly to the field of computational biology but also opens new horizons for drug repurposing and the development of innovative approaches to combat microbial infections. It underscores the need for an interdisciplinary approach, integrating computational biology, traditional medicine, and molecular biology, to discover novel solutions to healthcare’s most pressing challenges. Future research should focus on validating these computational findings through *in vitro* and *in vivo* studies, advancing our understanding of these phytochemicals’ therapeutic potential and their role in sustainable microbial management.

## 5 Conclusion and future prospects

The present research has delineated significant advancements in our comprehension of the synergistic interplay between curcumin and mangiferin, particularly focusing on their binding affinities with the bacterial transpeptidase enzyme CapD. Employing cutting-edge computational techniques, the study elucidates potential therapeutic pathways, offering a compelling argument for the repurposing of these phytochemicals in the medical armamentarium against bacterial infections, notably anthrax. This work contributes not only to the scientific landscape but also provides a rigorously validated framework for the integration of traditional medicinal knowledge with modern pharmacological paradigms.

As the scientific community ponders future research vectors emanating from this work, several critical avenues necessitate exploration. Firstly, it is imperative to transition from computational predictions to empirical validation. This will necessitate a robust interdisciplinary framework that seamlessly integrates microbiology, pharmacology, and computational biology for an unequivocal understanding of both the therapeutic potential and safety profile of the investigated compounds. Secondly, gaining a granular understanding of the underlying molecular mechanisms that drive the observed synergistic effects between curcumin and mangiferin becomes imperative. This is indispensable for rational drug design, aimed at maximizing efficacy, improving target specificity, and minimizing adverse effects.

Finally, from a global healthcare vantage, this research stands as a crucial milestone in the escalating fight against antimicrobial resistance. It holds the potential to serve as a cornerstone for multi-institutional, international collaborations aiming to develop sustainable, effective pharmacological interventions against microbial pathogens.

## Data availability statement

The original contributions presented in the study are included in the article/Supplementary material, further inquiries can be directed to the corresponding authors.

## Author contributions

KP: Conceptualization, Data curation, Project administration, Resources, Writing – original draft, Writing – review & editing. NB: Data curation, Formal analysis, Investigation, Writing – original draft, Writing – review & editing. NA: Data curation, Formal analysis, Methodology, Writing – original draft. SK: Data curation, Formal analysis, Investigation, Writing – original draft. JA: Data curation, Formal analysis, Investigation, Methodology, Resources, Validation, Writing – original draft, Writing – review & editing. SD: Conceptualization, Data curation, Formal analysis, Investigation, Methodology, Resources, Supervision, Validation, Writing – original draft, Writing – review & editing. VK: Conceptualization, Data curation, Funding acquisition, Resources, Writing – original draft, Writing – review & editing.
